# Systemic Copper Chelation Reduces Collagen Deposition and Preserves Secretory Function in Irradiated Mouse Salivary Glands

**DOI:** 10.1016/j.adro.2026.102149

**Published:** 2026-07-22

**Authors:** Kihoon Nam, Frank M. Maslow, Harim Tavares Dos Santos, Vinit C. Shanbhag, Rodrigo A. Osorio, Michael J. Petris, Olga J. Baker

**Affiliations:** a Christopher S. Bond Life Sciences Center, University of Missouri, Columbia, Missouri; b School of Medicine Department of Otolaryngology-Head and Neck Surgery, University of Missouri, Columbia, Missouri; c School of Dental Medicine, Department of Oral Biology, University at Buffalo, The State University of New York, Buffalo, New York; d Department of Biochemistry, University of Missouri, Columbia, Missouri; e Department of Ophthalmology, University of Missouri, Columbia, Missouri

## Abstract

**Purpose:**

Radiation therapy (RT) for head and neck cancer commonly causes fibrosis of the salivary gland (SG) and loss of secretory function. We previously demonstrated that transdermal injection of the copper chelator tetrathiomolybdate into irradiated submandibular glands reduced the early deposition of fibrillar collagen and preserved their integrity and function. Although these studies identified copper metabolism as a novel therapeutic target to control RT-induced damage to the SG, local delivery of copper chelators in human SG presents clinical hurdles including specialized personnel and equipment needed for administration together with the discomfort felt by patients upon receiving this treatment. To overcome these issues, the current study investigated whether systemic administration of tetrathiomolybdate (TTM) via drinking water could similarly reduce collagen deposition and preserve secretory function.

**Methods and Materials:**

Mice received TTM in drinking water (0.1 mg/mL) beginning 7 days before and continuing through 7 days after a single 15-Gy dose of RT to the neck. Systemic TTM exposure and copper bioavailability were assessed by measuring molybdenum and copper levels and serum ceruloplasmin activity. RT-induced SG injury was evaluated by collagen deposition, histopathology, epithelial integrity, and stimulated saliva secretion.

**Results:**

Systemically administered TTM reached the submandibular gland and reversibly reduced copper bioavailability, as demonstrated by increased molybdenum levels, reduced copper-to-molybdenum ratios, and suppression of serum ceruloplasmin activity. Moreover, TTM treatment attenuated RT-induced collagen deposition and epithelial damage and promoted recovery of ZO-1 expression. Finally, at 30 days after RT, stimulated saliva flow was significantly higher in TTM-treated irradiated mice than in irradiated mice receiving regular water.

**Conclusion:**

Systemic TTM administration reduces copper bioavailability, attenuates RT-induced collagen deposition and epithelial injury, and preserves SG secretory function. These findings support systemic copper chelation as a less invasive alternative to local TTM delivery for limiting RT-induced SG damage.

## Introduction

Radiation therapy (RT) for head and neck cancer causes fibrosis of the salivary glands (SGs), leading to hyposalivation.^1^, ^2^, ^3^ An early step in fibrosis is the enzymatic oxidation of lysine and hydroxylysine residues in collagen via the lysyl oxidase (LOX) family of copper-dependent metalloenzymes.^3^, ^4^, ^5^ This oxidative reaction causes formation of lysine aldehydes, which create covalent bonds with other collagen molecules, resulting in collagen crosslinking.^6^^,^^7^ Given that copper is a cofactor for all LOX family members, regulating copper metabolism presents a potential therapeutic target for managing RT-induced fibrosis in SG.^8^, ^9^, ^10^ To this end, a previous study demonstrated that transdermal injection of the copper chelator tetrathiomolybdate (TTM) into the submandibular glands (SMGs) significantly reduces collagen deposition in irradiated tissues while maintaining organ integrity and function.^11^ Although these findings highlight the potential of repurposing clinically approved copper chelators as neoadjuvant treatments for RT,^12^^,^^13^ transdermal injection in humans poses significant clinical hurdles including specialized personnel and equipment needed for administration^14^ together with the discomfort felt by patients upon receiving this treatment. To overcome the issues of local administration of TTM in irradiated human SG, systemic administration appears to be a potentially superior approach, provided that the treatment gains remain. Systemic delivery of TTM via an oral route has proven effective and well tolerated among patients with Wilson’s disease (ie, a rare inherited condition that causes copper levels to build up in several organs, especially the liver, brain, and eyes)^15^, ^16^, ^17^ without the concerns noted earlier. Therefore, the current study investigated whether systemic administration of TTM via an oral route (ie, in drinking water) could elicit results comparable to those seen with transdermal injection in terms of reduced collagen deposition and restoration of functionality,^11^ thereby demonstrating the effectiveness of this more convenient and patient-friendly application while retaining the treatment benefits of the relatively more complex and invasive method employed earlier.^11^

## Methods and Materials

### Materials

Ammonium tetrathiomolybdate (TTM), pilocarpine, goat serum, xylene, Tween 20, fluorescein sodium salt, hematoxylin and eosin (H&E) Y solution were purchased from MilliporeSigma. Ethanol was purchased from Decon Laboratories. Tris-EDTA buffer, Picro Sirius Red Stain Kit and 4′,6-diamidino-2-phenylindole (DAPI) were purchased from Abcam. Triton X-100, phosphate buffered saline (PBS), xylene-based mounting solution and rabbit anti-zona occludens 1 (ZO-1) were purchased from Thermo Fisher Scientific. Mouse anti-E-cadherin was purchased from Becton, Dickinson and Company (BD) Biosciences. Alexa Fluor 488 conjugated anti-rabbit immunoglobulin G (IgG) secondary antibody and Alexa Fluor 568 conjugated anti-mouse IgG secondary antibody were purchased from Invitrogen.

### Animals

Female C57BL/6J mice (8-week-old, 18-20 g) were purchased from Jackson Laboratory and allowed to acclimate for 2 weeks. Mice were maintained under standard housing conditions with a 12 hour light/12 hour dark cycle and provided food and water ad libitum. The animals’ behavior, food intake, signs of pain, and overall health were evaluated daily by a veterinarian who was not directly involved in the experiment. This independent care and monitoring ensured that the experimental results were not influenced by the animal management process. Procedures were performed during the morning to maintain consistency across experiments with no bolus material applied during the RT. Moreover, quality assurance methods for laboratory mice were performed to ensure the health, genetic purity as well as animal welfare. All procedures followed the Animal Research: Reporting *In Vivo* Experiments (ARRIVE) guidelines, with no adverse effects observed. All animal procedures, including anesthesia and euthanasia, were conducted in accordance with the Animal Care and Use Committee (ACUC protocol #46402) at the University of Missouri.

### Systemic administration of TTM via drinking water

**Figure 1:**
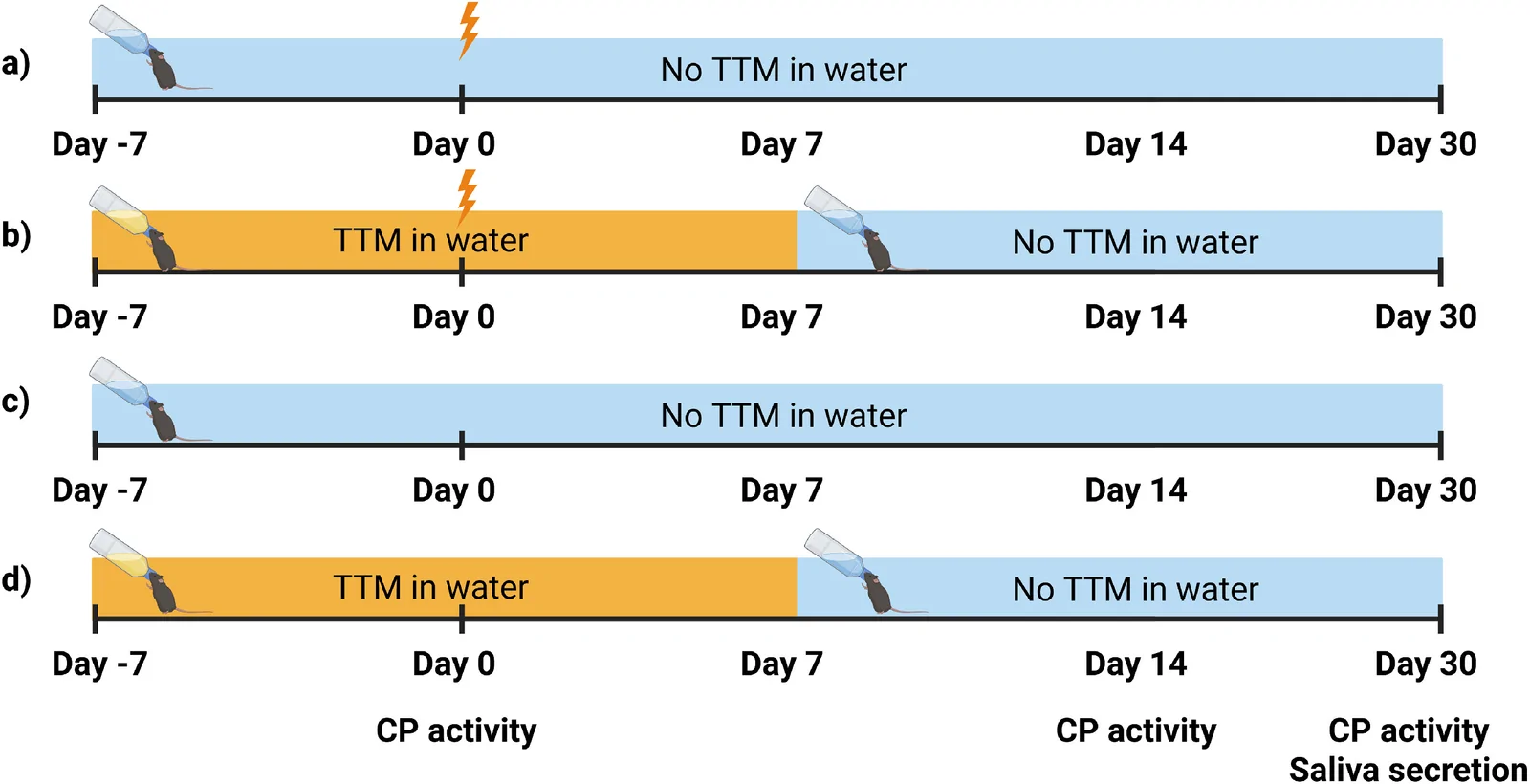
Experimental design of radiation therapy and systemic TTM administration. Eight-week-old mice were initially randomized into 2 drinking groups: regular water (blue) or TTM-treated water (yellow) beginning at day −7. Each drinking group was further subdivided into RT and non-RT cohorts at day 0 thereby generating 4 experimental groups: a) RT drinking regular water, b) RT drinking TTM water, c) non-RT drinking regular water and d) non-RT drinking TTM water. For RT studies, a single 15 Gy dose was delivered to the neck region at day 0 using a customized lead shield with a 1-cm × 4-cm slit opening. TTM-treated mice received TTM in drinking water from day −7 to day +7, after which all mice were maintained on regular water. Tissues were then harvested at days 14 and 30 post-RT and saliva collected at day 30. *Abbreviations:* RT = radiation therapy; TTM = tetrathiomolybdate.

For systemic administration, mice were initially randomized into 2 drinking treatment groups based on prior studies in which oral TTM was given at 1 mg per day to deplete copper in mice^18^: (1) regular water and (2) TTM-treated water (0.1 mg/mL, Fig. 1). Treatment began 7 days prior to RT (day −7) and continued through 7 days post-RT (day +7). Because TTM is light-sensitive, water bottles were wrapped in aluminum foil and replaced weekly. Based on average daily consumption (∼2.3 mL per mouse), the estimated TTM intake was approximately 230 µg per day (Fig. E1). To confirm systemic exposure, molybdenum levels were measured in both serum and SMG at day 0 (ie*,* the day of RT).

### Radiation therapy

Mice from each drinking group were subdivided into RT and non-RT groups at day 0, resulting in 4 experimental groups: (a) RT drinking regular water; (b) RT drinking TTM water; (c) non-RT drinking regular water; and (d) non-RT drinking TTM water (Fig. 1). The sample size per group was determined using G*Power software (v 3.1.9.7) with a power of 0.95, a significance level of 0.05 and an effect size (f) of 1.1 totaling n = 20 mice per group. Mice were anesthetized with 2.5% isoflurane (100 mL/min) and fitted with a protective lead shield with a customized ventral opening of 1 cm × 4 cm above the neck region at day 0. Next, mice were exposed to a single 15-Gy (X-ray) radiation dose using the Precision X-Ray MultiRad225 cabinet irradiator (Precision X-Ray) delivered at 0.348 Gy/second and cohorts from all treatment groups were analyzed at days 14 and 30 post-RT. The irradiator was operated at 225 kVp and 17.8 mA with animals positioned on shelf number 6, corresponding to a source-to-surface distance of 22 cm. A fixed circular collimator was used thereby providing a radiation field diameter of 14.5 cm. Moreover, beam filtration consisted of a 0.5-mm aluminum filter with dose verification performed using an ionization chamber according to the manufacturer’s protocol with the most recent calibration completed prior to the initiation of RT.

### Measurement of molybdenum and copper levels

Molybdenum and copper levels were measured in serum and SMG, respectively, using Inductively Coupled Plasma Mass Spectrometry (ICP-MS), as previously described.^19^ Briefly, specimens were digested overnight in 50% nitric acid, and metal ion content was determined using PlasmaQuad (PQ) ExCell ICP-MS. Serum molybdenum levels were expressed in parts per billion (ppb), whereas copper levels in SMG were normalized to wet tissue weight and expressed as mg/kg thereby allowing copper/molybdenum ratios to be calculated with each group yielding a total of 4 biological replicates.

### Measurement of ceruloplasmin activity

Blood samples were collected on days 0 and 14 using a cardiac micropuncture technique.^20^ Samples were allowed to clot at room temperature for 5 min and then centrifuged at 13,000 × g for 10 minutes at 4 °C to obtain serum. Ceruloplasmin activity, used as a biomarker of bioavailable copper, was determined by measuring the rate of *o*-dianisidine oxidation, as previously described.^21^^,^^22^ Briefly, serum samples were incubated at 37 °C and absorbance at 540 nm was recorded at 5 and 60 minutes. Finally, oxidase activity (units/L) was calculated using the formula: (A_60_ − A_5_) × 113.6. Each sample was measured in triplicate technical replicates per mouse (n = 4 biological replicates per group; total n = 12 technical replicates). Data are presented as mean ± SD with statistical comparisons between groups performed at day 0 using an unpaired 2-tailed Welch’s *t*-test in GraphPad Prism (v10).

### Picro Sirius Red staining

Four mice per group were randomly selected and euthanized at days 14 and 30 post-RT. Next, SMG were dissected and fixed in 10% formalin at room temperature for 24 hours and dehydrated in 70% ethanol solution at 4 °C for an additional 24 hours. Then, specimens were dehydrated in 100% ethanol, embedded in paraffin wax and cut into 5-μm sections. The sections were deparaffinized with xylene and rehydrated with serial ethanol solutions of 100%, 95%, 80%, 70%, and 50% (v/v) followed by a rinse with distilled water. For collagen staining, the rehydrated sections were stained with Picro Sirius Red for 1 hour and washed twice with acetic acid solution (0.5%, v/v). Then, slides were rinsed with absolute ethanol, cleared in xylene and mounted with a xylene-based mounting solution. Brightfield picrosirius red (PSR) images were captured using a Leica DMI6000B microscope (Leica Microsystems), whereas second harmonic generation (SHG) imaging was performed using a Leica SP8 laser scanning confocal microscope (Leica Microsystems) to visualize fibrillar collagen architecture.^23^ Regions of interest were restricted to the SMG, while signals originating from adjacent structures (eg, sublingual gland tissue, peripheral gland borders, and surrounding interglandular vasculature) were excluded algorithmically. SHG images were subsequently analyzed using artificial intelligence-guided image analysis software (AIVIA) to visualize relative collagen-associated signal intensity and quantify collagen-positive area (%) within the defined regions of interest.^11^ Collagen-positive area was calculated as the percentage of collagen-positive pixels relative to the total SMG area across biological replicates.

### Hematoxylin and eosin staining

For H&E staining, the rehydrated sections were treated with hematoxylin for 5 minutes and rinsed twice with distilled water to remove any excess stain. Bluing reagent was then applied to the tissue sections with a 10-second incubation followed by rinsing twice with distilled water. Then, the slides were dipped in 100% ethanol, excess ethanol was removed and eosin Y solution was applied to the tissue sections for 3 minutes. Slides were then rinsed, dehydrated using absolute ethanol, cleared and mounted in a xylene-based mounting solution and images were captured using the Leica DMI6000B fluorescent microscope under bright field setting. H&E analysis was performed to provide representative histopathological context and was not intended as a quantitative endpoint.

### Confocal immunofluorescence analysis

For antigen retrieval, the rehydrated SMG sections were immersed in Tris-EDTA buffer [10 mM Tris, 1 mM EDTA, 0.05% (v/v) Tween 20, pH 9.0] and processed using a digital electric pressure cooker (Nesco Professional) under the manufacturer-defined “high” pressure setting (approximately 10-12 psi, ∼115-121 °C) for 20 minutes after reaching full pressure. The total processing time, including heating and cooling, was approximately 40 minutes. Next, samples were permeabilized with 0.1% (v/v) Triton X-100 in PBS at room temperature for 45 minutes. Specimens were then blocked in 5% (v/v) goat serum in PBS for 1 h at room temperature and incubated at 4 °C overnight with the following primary antibodies: rabbit anti-ZO-1 (1:1000), mouse anti-E-cadherin (1:100). Later, sections were incubated with Alexa Fluor 488 (green) conjugated anti-rabbit IgG secondary antibody (1:500) and Alexa Fluor 568 (red) conjugated anti-mouse IgG secondary antibody (1:500) at room temperature for 1 hour and counterstained with 300 nM DAPI (blue) at room temperature for 5 minutes and mounted using ProLong Gold Antifade Mountant. Subsequently, specimens were imaged using the STELLARIS 5 Confocal Microscope (Leica Microsystems) with a 20 × objective where 5 randomly selected nonoverlapping fields per mouse were captured by an investigator blinded to treatment allocation. ZO-1 positive pixels were quantified for each treatment condition using ImageJ 1.52p and data expressed as the percentage of positive area relative to total tissue area. Data from each mouse represent the mean ± SD of results from a total of 4 mice per group and analyzed using 1-way analysis of variance followed by Tukey’s posthoc test for multiple comparisons in GraphPad Prism (v10).

### Saliva secretion measurements

Mice treated with regular water (control group) or TTM water (treatment group) at day 30 post-RT were anesthetized with Avertin solution followed by intraperitoneal injection with pilocarpine (2.5 mg/kg) to stimulate saliva secretion, which was collected over a 20-minute period and measured using a 200-μL pipette. Next, the normalized saliva flow rate was calculated using the following equations:

$$\text{Saliva}\;\text{flow}\;\text{rate}=\;\frac{\text{Stimulated}\;\text{saliva}\;\text{volume}\;\left(\mu L\right)\;}{\text{Mouse}\;\text{body}\;\text{weight}\;\left(g\right)\;\times \;\text{Collection}\;\text{time}\;\left(\text{min}\right)}$$

Statistical significance was assessed using 1-way analysis of variance (*P* ≤ .05) followed by Dunnett’s posthoc test using GraphPad Prism (v 10) software.

## Ethics statement

The study was conducted with the approval of the University of Missouri Animal Care and Use Committee. Also, this research adhered to the Animal Research: Reporting *In Vivo* Experiments (ARRIVE) guidelines, ensuring ethical animal treatment and research reporting standards.

Radiation therapy (RT) is a common treatment for head and neck cancer; however, it typically leads to fibrosis of the salivary gland (SG) with consequent loss of function. A previous study demonstrated that transdermal injection of tetrathiomolybdate into irradiated submandibular glands reduced the early deposition of fibrillar collagen and preserved their integrity and function. Although these studies identified copper metabolism as a novel therapeutic target to control RT-induced damage to the SG and the therapeutic potential of repurposing clinically approved copper chelators as neoadjuvant treatments for RT, local delivery of copper chelators in human SG presents clinical hurdles including specialized personnel and equipment needed for administration together with the discomfort felt by patients upon receiving this treatment. To overcome these issues, the current study investigated whether systemic administration of tetrathiomolybdate via drinking water reduces collagen deposition and preserves secretory function in irradiated mouse SG, consistent with earlier findings obtained when using local delivery, in an aim to test the functional equivalency of these 2 treatment modalities.

## Results

### Systemic TTM treatment reduces copper bioavailability in SMG following RT

**Figure 2:**
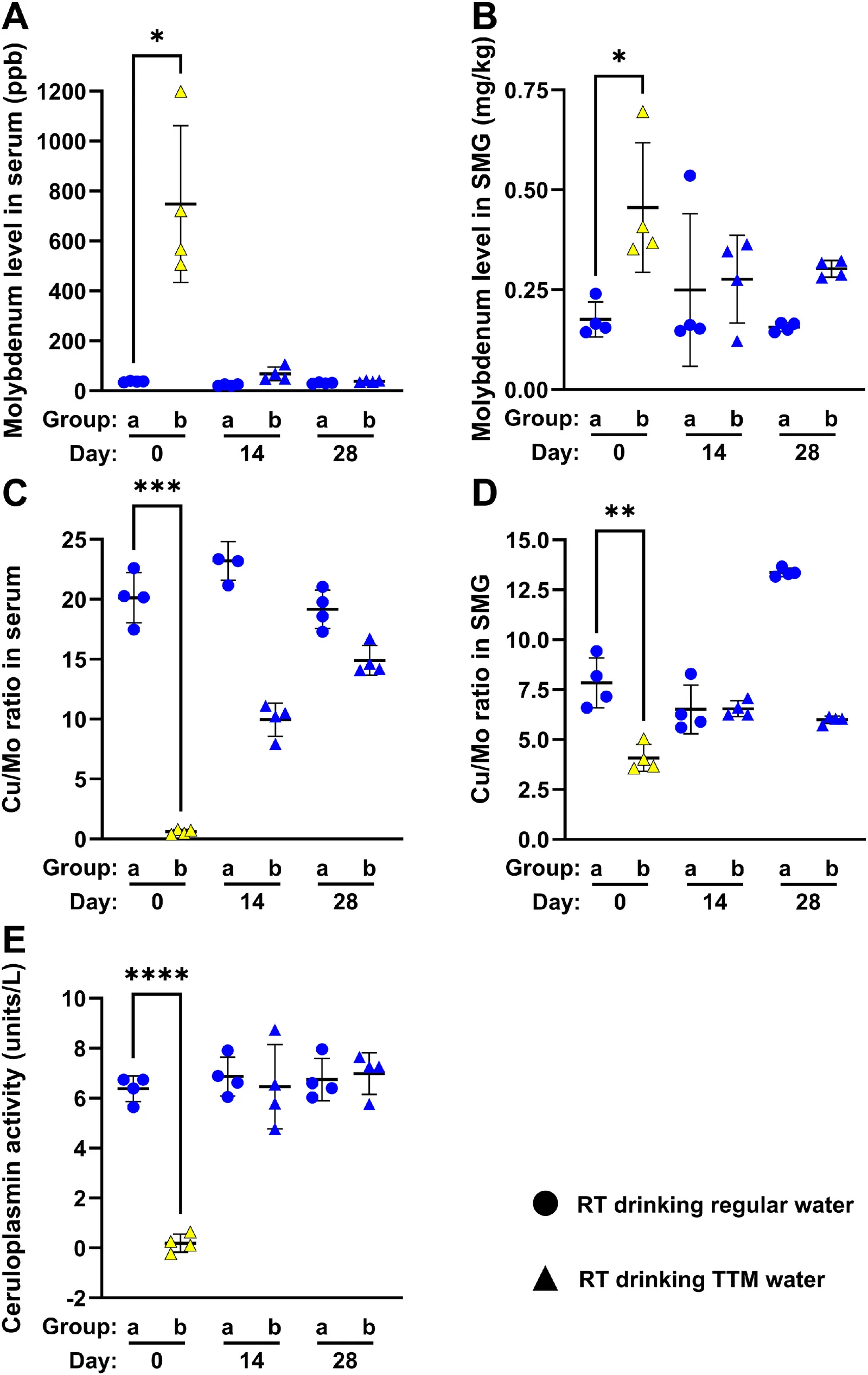
Systemic TTM treatment in drinking water reduces copper bioavailability. Molybdenum and copper levels were measured in serum and SMG, respectively, using Inductively Coupled Plasma Mass Spectrometry (ICP-MS). Specimens were digested with 50% nitric acid overnight and metal ions were measured by PlasmaQuad (PQ) ExCell ICP-MS. Moreover, molybdenum levels in serum were expressed as parts per billion (ppb) (A) whereas levels in SMG were expressed as mg/kg wet tissue weight (B). Additionally, copper/molybdenum ratio in serum (C) and SMG (D) was calculated from these measurements (n = 4 biological replicates per group). Finally, ceruloplasmin activity in serum was measured using o-dianisidine dihydrochloride as a substrate with triplicate samples incubated at 37 °C, absorbance recorded at 540 nm after 5 and 60 minutes, whereas oxidase activity (units/L) was calculated using the following formula: (A_60_−A_5_) × 113.6 (E). For Ceruloplasmin (CP) activity, triplicate technical replicates were performed per mouse and averaged to generate 1 value per mouse (n = 4 biological replicates per group). Circles represent RT mice drinking regular water (group a) and triangles represent RT mice drinking TTM water (group b). Yellow triangles indicate mice actively receiving TTM drinking water at day 0 while blue triangles indicate removal of TTM water. Therefore, all mice were maintained on regular water until the experimental points at days 14 and 30. Data are expressed as mean ± SD of results from a total of 4 mice per group with comparisons between groups (a and b) performed only at day 0 using an unpaired 2-tailed Welch’s *t*-test given that TTM administration was ongoing at this time point (*P < .05, **P < .01, ***P < .001 and ****P < .0001). *Abbreviations:* RT = radiation therapy; SMG = submandibular gland; TTM = tetrathiomolybdate.

To determine TTM absorption and clearance, we measured molybdenum levels in serum using ICP-MS as described in Methods and Materials. Our results show that molybdenum levels are significantly increased in serum during TTM treatment to ∼800 ppb and return to basal levels of ∼30 ppb after the treatment is discontinued (*P* < .05, Fig. 2A). These results indicate that TTM is absorbed into the bloodstream during the treatment and rapidly cleared after treatment. Likewise, to evaluate TTM levels in the SMG, we measured molybdenum levels in SMG tissues, again using ICP-MS. Moreover, molybdenum levels significantly increase during TTM treatment to ∼0.456 mg/kg and rapidly return to pre-treatment levels of ∼0.176 mg/kg after the treatment is discontinued (Fig. 2B), with these findings further indicating that systemic treatment with TTM reaches the SMG and is rapidly cleared after treatment cessation. TTM has a high copper-binding affinity (10^−20^ M) which reduces both dietary copper absorption^24^^,^^25^ and its intracellular trafficking to metalloenzymes.^26^ Therefore, the copper-to-molybdenum ratio is a suitable indicator of “bioavailable” copper in tissues, and to this end, we measured the copper-to-molybdenum ratio in both serum and SMG as described in Methods and Materials. As shown in Fig. 2C and 2D, systemic TTM treatment significantly reduced the copper-to-molybdenum ratio in both serum by 97% (*P* < .001) and SMG by 48% (*P* < .01), respectively, with these results indicating that systemic TTM treatment reduces copper bioavailability in SMG during treatment. Ceruloplasmin is a copper-dependent oxidase secreted primarily by hepatocytes and accounts for more than 90% of total plasma copper.^27^ Because copper-free apoceruloplasmin is rapidly degraded,^28^^,^^29^ decreased ceruloplasmin oxidase activity in plasma is a well-established biomarker of systemic copper deficiency. In our study, plasma ceruloplasmin activity was nearly undetectable after 7 days of TTM administration (Fig. 2E, yellow triangles) but returned to baseline levels within 7 days following treatment cessation. Moreover, although TTM is known to undergo oxidation and ligand exchange reactions in aqueous environments, leading to changes in its chemical speciation,^30^^,^^31^ mice consuming TTM in the water remained copper depleted on day 7 as determined by significantly reduced serum ceruloplasmin activity. Together, these results indicate that oral TTM treatment reversibly reduced bioavailable copper both in the bloodstream and in the SMG during treatment.

### Systemic TTM treatment prevents collagen deposition in SMG following RT

**Figure 3:**
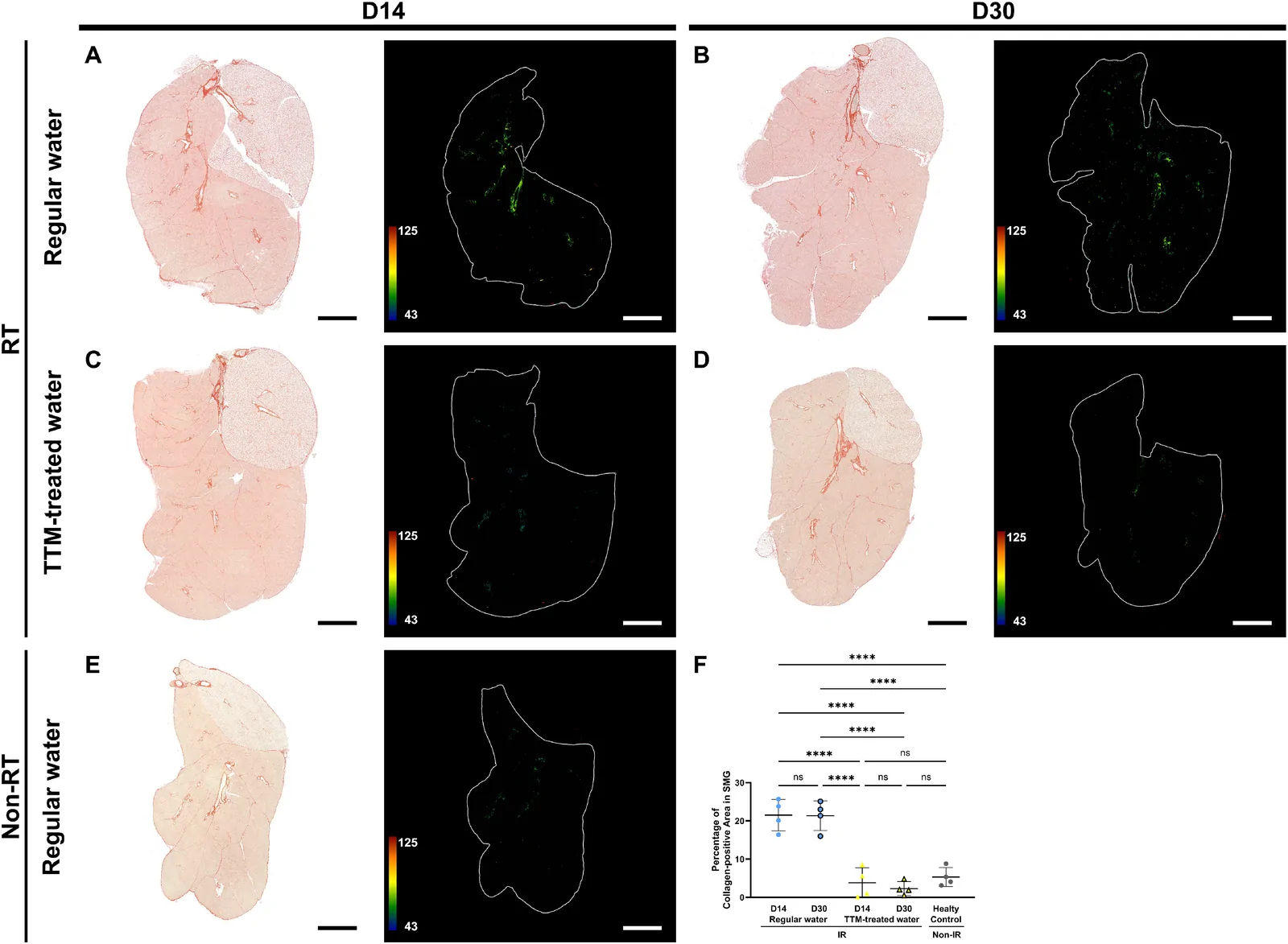
Systemic TTM treatment in drinking water prevents collagen deposition in SMG from irradiated mice. SMG tissue sections from mice within the following groups: RT drinking regular water at day 14 (A), RT drinking regular water at day 30 (B), RT drinking TTM water at day 14 (C), RT drinking TTM water at day 30 (D) non-RT drinking regular water (E) were stained with Picro Sirius Red and visualized using second harmonic generation (SHG) microscopy. Representative images were analyzed using AIVIA software, with color intensity indicating relative collagen-associated SHG signal and white outlines defining the SMG region of interest. Quantification of collagen-positive area (%) within the SMG was performed across biological replicates using AIVIA analysis and included images shown in Fig. E2. Data are expressed as mean ± SD (n = 4 mice/group) (F). Statistical significance was assessed using 1-way ANOVA followed by Tukey’s posthoc test (***P < .001, ****P < .0001, ns = not significant). Scale bars = 1 mm. *Abbreviations:* ANOVA = analysis of variance; RT = radiation therapy; SMG = submandibular gland; TTM = tetrathiomolybdate.

To determine whether collagen deposition in irradiated SMG can be prevented by systemic copper chelation, mice were treated in the absence (ie, regular drinking water) or presence of TTM (ie, TTM added to drinking water) with a total of 4 treatment groups: (a) RT drinking regular water; (b) RT drinking TTM water; (c) non-RT drinking regular water; and (d) non-RT drinking TTM water. Subsequently, SMG tissue sections were stained with Picro Sirius Red and analyzed as described in Methods and Materials. Results showed that SMG from mice in the RT + regular water group at days 14 and 30 post-RT exhibited stronger collagen-associated SHG signal intensity compared with non-RT controls (Fig. 3A-C), suggestive of increased collagen accumulation. Quantification of collagen-positive area (%) using AIVIA analysis across biological replicates, including representative images shown in Fig. E2, demonstrated a significant increase in collagen coverage in irradiated SMG receiving regular water (*P* < .0001, Fig. 3F). In contrast, irradiated mice receiving TTM-treated water exhibited reduced collagen-associated SHG signal intensity at both day 14 and day 30 post-RT (Fig. 3C, D), with no significant differences in collagen-positive area compared with non-irradiated controls (Fig. 3F). These findings indicate that systemic TTM treatment attenuates RT-induced collagen deposition in SMG.

### Systemic TTM treatment prevents epithelial damage in SMG following RT

**Figure 4:**
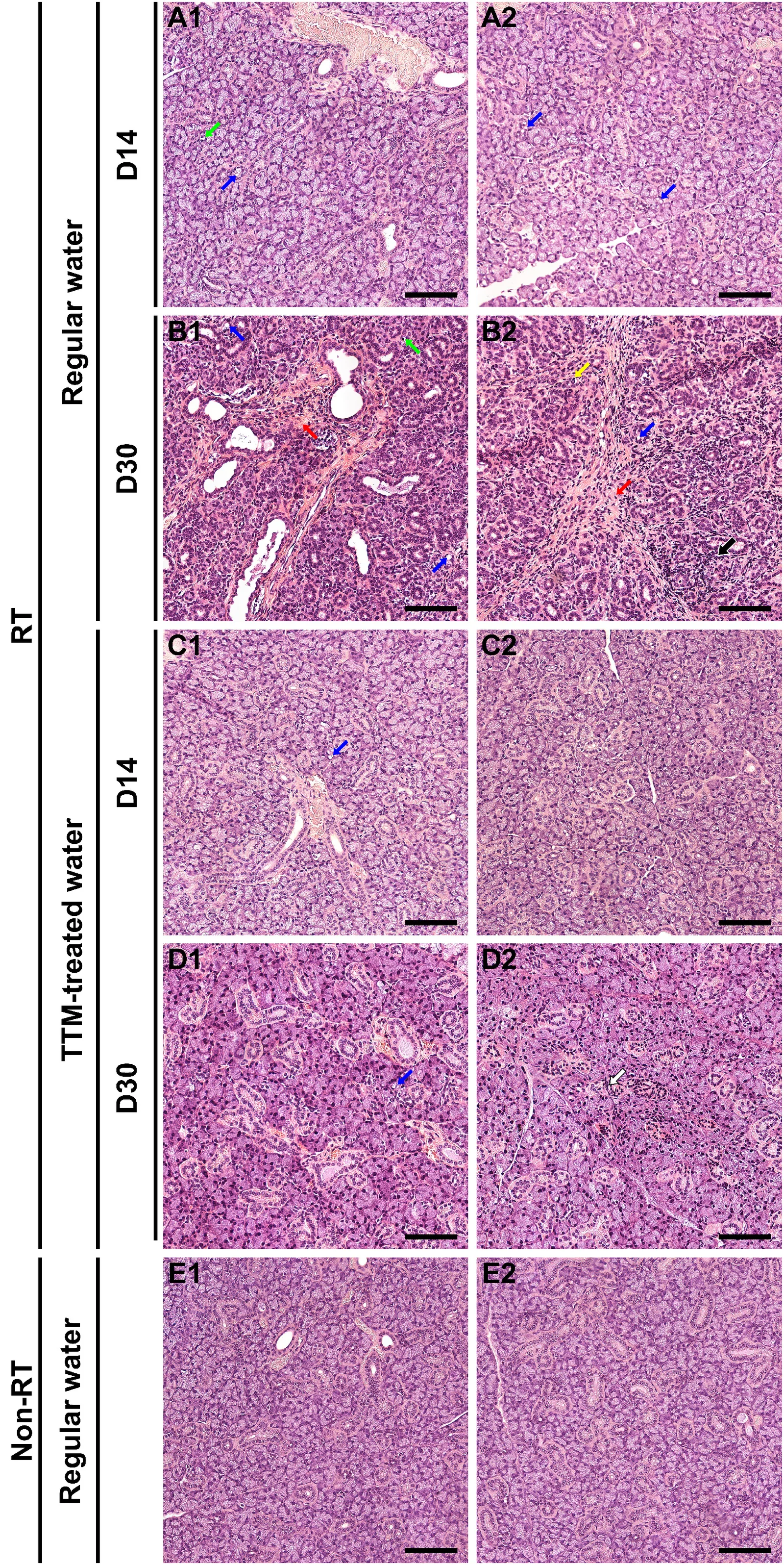
Systemic administration of TTM via drinking water preserves tissue architecture in irradiated SMG. SMG tissue sections from mice within the following groups: RT drinking regular water at day 14 (A1-2), RT drinking regular water at day 30 (B1-2), RT drinking TTM water at day 14 (C1-2), RT drinking TTM water at day 30 (D1-2), non-RT drinking regular water (E1-2) were stained with Hematoxylin and eosin-stained and sections analyzed using a Leica DMI6000B microscope. Arrows indicate representative histopathological features, including focal areas of cell degeneration (green), cytoplasmic vacuolization (blue), excessive accumulation of connective tissue (red), round cells with condensed cytoplasm and fragmented purple nuclear material (yellow), features suggestive of inflammatory cell infiltration (black), abnormal nucleus and cytoplasm morphology (white). Images are representative of a total of 4 mice per group with scale bars set at 50 μm. *Abbreviations:* RT = radiation therapy; SMG = submandibular gland; TTM = tetrathiomolybdate.

To provide complementary histopathological assessment of tissue morphology following RT, SMG tissue sections from treated mice were stained with H&E. Our results show that SMG from mice within the RT drinking regular water group at day 14 post-RT displayed focal areas of cell degeneration (Fig. 4A1, green arrow) and empty, clear spaces consistent with cytoplasmic vacuolization (Fig. 4A1, A2, blue arrows) suggestive of early epithelial tissue damage. Moreover, mice drinking regular water at day 30 post-RT displayed focal areas of cell degeneration (Fig. 4B1, green arrow), excessive accumulation of connective tissue suggestive of fibrosis (Fig. 4B1, B2, red arrows), presence of round cells with condensed cytoplasm and fragmented purple nuclear material, suggestive of apoptosis (Fig. 4B2, yellow arrow), together with features suggestive of inflammatory cell infiltration (Fig. 4B2, black arrow), all of which are consistent with late epithelial tissue damage in irradiated SMG. In contrast, SMG from mice within the RT drinking TTM water at day 14 post-RT displayed limited cytoplasmic vacuolization (Fig. 4C1, blue arrow) and absence of cell degeneration (Fig. 4C1, C2), suggesting attenuation of early epithelial tissue alterations following systemic TTM treatment. Finally, although SMG from mice within the RT drinking TTM water at day 30 showed modest signs of RT-mediated tissue damage (eg, a few points of cell degeneration as noted in Fig. 4D1, blue arrow) and a sporadic area with abnormal nucleus and cytoplasm morphology suggestive of cell atypia as noted in Fig. 4D2, white arrow; however, the overall tissue architecture was consistent with non-RT drinking regular water group at this time point (Fig. 4E1, E2), with tiled images providing confirmatory analysis shown in Fig. E3A-E.

**Figure 5:**
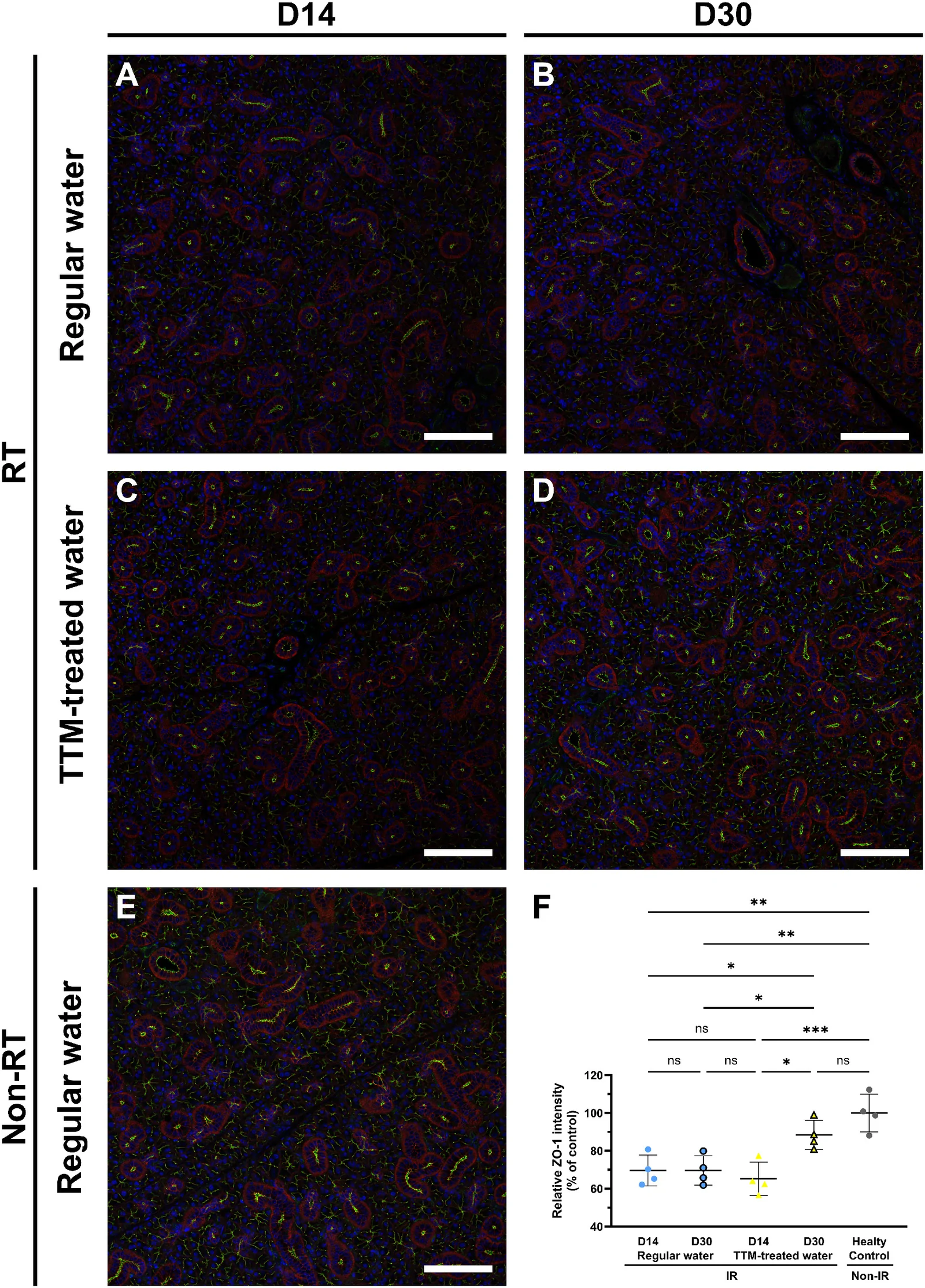
Systemic TTM treatment in drinking water preserves ZO-1 expression in SMG from irradiated mice. SMG tissue sections from mice within the following groups: RT drinking regular water at day 14 (A), RT drinking regular water at day 30 (B), RT drinking TTM water at day 14 (C), RT drinking TTM water at day 30 (D), non-RT drinking regular water (E) were analyzed using confocal microscopy with specific antibodies against ZO-1 [green; (A-E)], E-cadherin [red; (A-E)] and counterstained with DAPI (blue; everywhere), as described in Methods and Materials. ZO-1 positive pixels were quantified for each treatment condition. Data are expressed as mean ± SD of results from a total of 4 mice per group (F). Statistical significance was assessed using 1-way ANOVA (*P < .05, **P < .01, ***P < .001 and ns = not significant) and Tukey’s posthoc test for multiple comparisons with scale bars set at 100 μm. *Abbreviations:* ANOVA = analysis of variance; RT = radiation therapy; SMG = submandibular gland; TTM = tetrathiomolybdate.

To evaluate whether epithelial polarity in irradiated SMG can be reduced by using systemic copper chelation, we analyzed the spatial organization of the apical tight junction protein ZO-1 and the basolateral adherens junction protein E-cadherin using confocal microscopy, as described in Methods and Materials. Results show that SMG from mice within the RT drinking regular water group at days 14 and 30 post-RT (Fig. 5A, B), as well as RT drinking TTM water at day 14 (Fig. 5C), displayed a significant reduction of ZO-1 expression when compared to SMG from mice within the non-RT drinking regular water group (*P* < .01, Fig. 5F). In contrast, SMG from mice within the RT drinking TTM water group at day 30 post-RT (Fig. 5D) displayed significantly higher ZO-1 expression compared to the RT drinking regular water groups at days 14 and 30 (*P* < .05, Fig. 5F). These results indicate that TTM attenuates RT-induced alterations in tight junction expression in irradiated SMG. Finally, E-cadherin expression and organization were unchanged across the groups studied (Fig. 5A-E), indicating no notable effects of RT or TTM on adherens junction expression under the conditions examined. Taken together, these qualitative histopathological observations complement the quantitative findings demonstrating reduced collagen deposition, preservation of epithelial integrity (ZO-1), and improved SG function following systemic TTM treatment.

### Systemic TTM treatment preserves saliva secretion in SMG following RT

**Figure 6:**
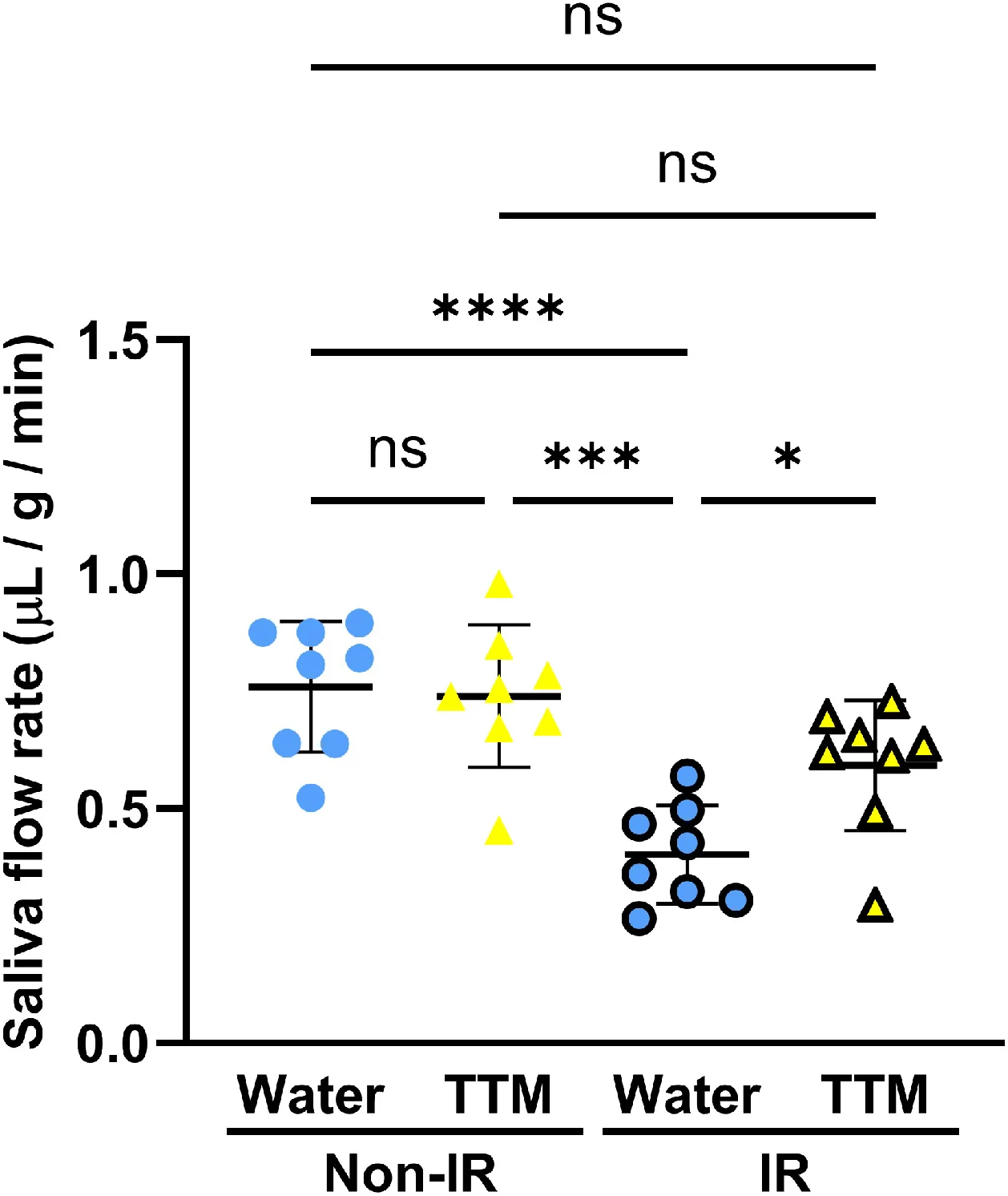
Systemic TTM treatment in drinking water prevents loss of saliva secretion in irradiated mice. At day 30 post-RT therapy, mice were anesthetized and stimulated with pilocarpine and saliva collected for 20 minutes. Data represent the mean ± SD of results from a total of 8 mice per group. Statistical significance was assessed using 1-way ANOVA (*P < .05, ***P < .001, ****P < .0001 and ns = not significant) and Tukey’s posthoc test for multiple comparisons. *Abbreviation:* ANOVA = analysis of variance, RT = radiation therapy; SMG = submandibular gland; TTM = tetrathiomolybdate.

Previous studies showed that the time point where more than 50% reduction of saliva secretion occurs after RT is approximately 30 days,^32^ and with this in mind, we compared saliva secretion in mice treated with TTM water versus those treated with regular water at day 30 post-RT. As shown in Fig. 6, in irradiated mice, the saliva flow rate was significantly higher in the TTM-treated group compared with the water-treated control group (*P* = .0412). In contrast, the difference in saliva flow rate between irradiated mice treated with TTM and nonirradiated mice treated with water was not statistically significant (*P* = .0839). Taken together, these findings suggest that TTM treatment adequately protects the SMG against RT-induced hyposalivation and is consistent with historical strain data.^11^^,^^32^

## Discussion

Although specifics regarding the mechanisms involved on a comparative basis (ie, relative reduction of collagen deposition and restoration of SG functionality for local vs systemic delivery of TTM) are beyond the scope of this current work, the following may be of benefit to elucidate the treatment pathways noted thus far. Specifically, the antifibrotic effects observed with systemic TTM likely reflect multiple mechanisms linked to the essential role of copper in redox biology and extracellular matrix remodeling.^33^^,^^34^ One plausible mechanism involves inhibition of the copper-dependent enzyme lysyl oxidase (LOX) and its isoforms (LOXL1-4), which catalyze the oxidative deamination of lysine and hydroxylysine residues in collagen and elastin, initiating crosslink formation and matrix stiffening.^19^^,^^35^^,^^36^ By reducing the copper bioavailability, TTM may suppress LOX activity, thereby limiting collagen crosslinking and the progressive matrix rigidity that drives fibroblast activation and tissue scarring.^37^, ^38^, ^39^ Indeed, copper chelation with TTM reduces LOX/LOXL expression and activity in experimental fibrosis models.^40^^,^^41^ Additionally, by reducing the bioavailability of redox-active copper, TTM may mitigate tissue injury caused by RT-induced production of reactive oxygen species.^12^^,^^42^^,^^43^ Emerging data suggest that TTM can also trigger autophagy-dependent activation of NRF2 and upregulation of antioxidant target genes (eg, *HMOX1, GCLM*) in vascular endothelial cells, offering protection against oxidative damage.^44^ Together, these cytoprotective responses may help preserve epithelial integrity in irradiated tissues, further reducing fibrotic remodeling. Despite this limited understanding of treatment mechanisms, the current findings support the feasibility of replacing the previous local administration method with a simpler, less invasive systemic approach, without compromising therapeutic benefits (eg, no significant differences noted in collagen deposition and saliva secretion between the 2 delivery methods).^11^

Several limitations were noted in this study. First, other markers of RT-mediated SG fibrosis (eg, proinflammatory cytokines transforming growth factorβ, interleukin-1, interleukin-6,^45^ and myofibroblast persistence)^46^ were not investigated here. Second, the lack of quantitative H&E assessment represents a limitation. However, H&E staining was intended to provide complementary histopathological context, whereas the principal study conclusions were based on quantitative assessments of collagen deposition, epithelial integrity (ZO-1), and SG function. Finally, variability in the volume of TTM-containing water consumed by individual mice may have influenced systemic exposure. Future studies using larger cohorts and direct monitoring of water intake would help address this limitation. Regarding the potential side effects of TTM, previous clinical studies in cancer patients administered TTM over the course of 2 years have reported that TTM is relatively safe and well tolerated, with neutropenia being the most common grade 3/4 toxicity affecting 3.7% of patients.^37^ We did not monitor adverse events in the mice in our studies which received TTM for 2 weeks, however it will be important to track such endpoints in future studies that evaluate the efficacy and safety of TTM across multiple doses and time points. That said, the current study has opened the way for justifiable use of systemically administered TTM in light of the positive preclinical effects observed in irradiated SG (ie, reduction of copper bioavailability together with prevention of collagen deposition and subsequent epithelial damage as well as maintaining saliva flow rates), with future studies warranted to better understand the general as well as comparative mechanisms, and ultimately, to perform long-term impact studies so that these findings may ease the suffering of head and neck cancer patients.

## Disclosures

Olga Baker reports financial support, administrative support, article publishing charges, and equipment, drugs, or supplies were provided by University of Missouri. Olga Baker reports financial support, administrative support, article publishing charges, and equipment, drugs, or supplies were provided by National Institute of Dental and Craniofacial Research. If there are other authors, they declare that they have no known competing financial interests or personal relationships that could have appeared to influence the work reported in this paper.

## Appendix Supplementary materials

Supplementary materials Image, application 1
