# Supplementary material for: Systemic Copper Chelation Reduces Collagen Deposition and Preserves Secretory Function in Irradiated Mouse Salivary Glands
Source: Adv Radiat Oncol. 2026 Jul 22;11(12):102149. doi: 10.1016/j.adro.2026.102149 (PMC13524675; doi:10.1016/j.adro.2026.102149)
Supplement: Supplementary file 1 [file mmc1.docx]

**Figure E1. Average daily intake of TTM-treated water in mice.** Water consumption was measured on days 3, 7, and 10 after treatment initiation (n = 10 mice per time point) and converted to daily intake values. On average, mice consumed 2.3 mL/day, corresponding to an approximate TTM intake of 230 µg/day. Data are presented as mean ± SD.

**Figure E2. Systemic TTM treatment in drinking water reduces collagen deposition in SMG from irradiated mice.** Picro Sirius Red-stained SMG tissue sections from irradiated mice that received regular water at day 14 and day 30 as well as TTM water at day 14 and day 30 were visualized with second harmonic generation microscopy using non-RT as healthy control and analyzed with an artificial intelligence-guided image analysis software (AIVIA) with scale bars set at 1 mm.

**Figure E3. Systemic administration of TTM via drinking water preserves tissue architecture in irradiated SMG.** Hematoxylin and eosin-stained SMG tissue sections from irradiated mice that received regular water at day 14 **(A)** and day 30 **(B)** as well as TTM water at day 14 **(C)** and day **30 (D)** were analyzed using a Leica DMI6000B microscope using non-RT SMG as healthy control **(E)**. Images are representative from a total of 4 mice per group with scale bars set at 1 mm. The red rectangles indicate the locations corresponding to the high magnification images in Figure 2, as described in the main text.
